# Draft Genome Sequence of Candida railenensis Strain CLIB 1423, Isolated from Papaya Fruit in French Guiana

**DOI:** 10.1128/mra.00554-22

**Published:** 2022-11-14

**Authors:** Hugo Devillers, Cécile Grondin, Angèle Thiriet, Jean-Luc Legras

**Affiliations:** a SPO, Univ Montpellier, INRAE, Institut Agro, Montpellier, France; University of California, Riverside

## Abstract

Here, we report the draft genome sequence and annotation of the yeast Candida railenensis strain CLIB 1423. The assembly consists of 57 nuclear scaffolds and 1 complete mitochondrial chromosome, for a total of 13.8 Mb (*N*_50_, 0.54 Mb; *L*_50_, 9). The annotation contains 6,013 coding DNA sequences (CDSs) (BUSCO completeness, 99.6%).

## ANNOUNCEMENT

Candida railenensis is an ascomycetous yeast from the family *Debaryomycetaceae*. It is closely related to the genus *Kurtzmaniella* (1). Only a few species from this clade of yeasts have been sequenced yet, while a recent study (2) stressed the critical need for new sequenced genomes to decipher the taxonomic relationships in the *Kurtzmaniella* species complex.

*C. railenensis* strain CLIB 1423 was isolated from a papaya fruit in French Guiana in 2010 during an extensive collection of yeasts performed by the Biological Resource Center CIRM—Levures (3). Cells were grown for 72 h at 28°C on liquid yeast extract-peptone-dextrose (YPD) medium. Genomic DNA was extracted following a phenol-chloroform extraction protocol. The quality of the genomic DNA was evaluated using spectrophotometry and electrophoresis on a 0.7% agarose gel. The prepared DNA was then sent to the iGenSeq sequencing platform (Brain Institute, Paris) for library preparation using the Nextera XT kit. The libraries were sequenced using the Illumina NovaSeq 6000 platform with the SP reagent kit (300 cycles), yielding 6,062,601 read pairs (paired-end [PE], 2 × 150-bp format; coverage, 132×).

The reads were trimmed using Fastp v0.23.1 (4) using default parameters, except that the minimal read length was set to 50 nucleotides (18% of reads were discarded). The cleaned reads were assembled using SPAdes v3.15.3 (5) with default parameters, considering the following kmers: 21, 33, 55, and 77. With a minimal length of 5 kb, 58 scaffolds were selected as the final assembly, representing a cumulative length of 13,800,509 bases, an average GC content of 39.16%, and a *N*_50_ value of 539,109 bases. This assembly consists of 57 linear nuclear scaffolds and 1 complete circular mitochondrial chromosome.

Structural annotation of the coding genes (CDSs) was performed using Augustus v3.4.0 (6) with the pretrained Debaryomyces hansenii data set as the reference, without additional data, limiting the intron length to 4 kb. This yielded 5,767 putative CDSs. Manual curation of these genes was performed with particular attention paid to genes containing introns, to detect spurious gene fusion. As a result, 6,013 CDSs were reported. The completeness of this annotation was evaluated using BUSCO v5.2.2 (7) with the saccharomycetes_odb10 lineage data set as the reference (2,137 proteins). Before curation, the BUSCO score was 98.6% (98.3% single copy). After curation, the BUSCO score reached 99.6% complete (99.3% single copy). Functional annotation of these 6,013 CDSs was performed using an annotation transfer tool that we are developing (https://github.com/hdevillers/go-fannot), which combines functional annotation retrieved from homology against the curated UniProt database (8) and motif detection using InterProScan (9).

In addition to protein coding genes, 222 tRNA genes were predicted using tRNAscan-SE v2.0.9 (10). A complete rRNA unit was identified in scaffold 43, as well as three isolated 5S rRNA features. The long and short subunit rRNAs of the mitochondria were annotated. Last, 6 small nuclear/nucleolar RNA features (U1 to U6) were reported in the annotation. All rRNA and noncoding RNA (ncRNA) features were identified using BLASTn (11) with reference features from the curated annotations of *D. hansenii* (GenBank accession number GCF_000006445.2) and Saccharomyces cerevisiae (GCF_000146045.2).

### Data availability.

The data are available under the BioProject accession number PRJEB51943. The BioSample accession number is SAMEA13788477. The raw sequencing data are available under the SRA accession number ERR9433495. The GenBank assembly accession number is GCA_935541525.1. The complete phenol-chloroform extraction protocol used is available at https://www.protocols.io/view/dna-extraction-protocol-5qpvorx47v4o/v1.
